# Comparison of three molecular assays for the detection and molecular characterization of circulating tumor cells in breast cancer

**DOI:** 10.1186/bcr3395

**Published:** 2013-03-07

**Authors:** Areti Strati, Sabine Kasimir-Bauer, Athina Markou, Cleo Parisi, Evi S Lianidou

**Affiliations:** 1 Analysis of Circulating Tumor Cells Lab, Lab of Analytical Chemistry, Department of Chemistry, University of Athens, University Campus, Athens, 15771, Greece; 2 Department of Gynecology and Obstetrics, University Hospital of Essen, University of Duisburg-Essen, Hufelandstrasse 55, Essen, D-45122, Germany

## Abstract

**Introduction:**

Comparison studies between different analytical methodologies for circulating tumor cells (CTC) detection and molecular characterization are urgently needed, since standardization of assays is essential before their use in clinical practice.

**Methods:**

We compared three different CTC molecular assays. To avoid discrepancies due to pre-analytical errors we used the same cDNAs throughout our study. CTC were isolated using anti-EpCAM and anti-MUC1 coated magnetic beads from 2 × 5 ml of peripheral blood of 254 early and 51 metastatic breast cancer patients and 30 healthy individuals. The same cDNAs were analyzed by: a) singleplex RT-qPCR assay for *CK-19*; b) multiplex RT-qPCR for *CK-19, HER-2, MAGE- A3*, and *PBGD*; and c) a commercially available molecular assay (AdnaTest BreastCancer) for *GA733-2, MUC-1, HER-2 *and *beta-actin*.

**Results:**

In early breast cancer, *CK-19 *RT-qPCR, multiplex RT-qPCR and the AdnaTest, were positive for the presence of CTC in 14.2%, 22.8% and 16.5% subjects, respectively. The concordance between the AdnaTest and *CK-19 *RT-qPCR was 72.4% while between the AdnaTest and multiplex RT-qPCR was 64.6%. In patients with overt metastasis, *CK-19 *RT-qPCR, multiplex RT-qPCR and the AdnaTest were positive in 41.2%, 39.2% and 54.9% patients, respectively. The concordance between the AdnaTest and *CK-19 *RT-qPCR was 70.6% while between the AdnaTest and multiplex RT-qPCR was 68.6%.

**Conclusions:**

All CTC assays gave similar results in about 70% of cases. Better agreement was found in the metastatic setting, possibly explained by the higher tumor load in this group. Discordances could be attributed to the different gene transcripts used to evaluate CTC positivity. Our results indicate the importance of CTC heterogeneity for their detection by different analytical methodologies.

## Introduction

Βlood testing for circulating tumor cells (CTC) has emerged as one of the hottest fields in cancer biomarkers research [1]. CTC enumeration is regarded as an early marker of response to systemic therapy and can be used as a liquid biopsy for repeated follow up examinations [2], while their molecular characterization could be translated to personalized targeted treatments [3,4]. CTC detection can give early information on relapse and disease progression [5-9], while individualized treatment strategies based on the molecular characterization of CTC could improve efficacy [10] and can predict early response to therapy [2,11]. There is increasing evidence of the potential benefits of using CTC detection in clinical practice, and until now more than 400 clinical studies have included CTC as a biomarker in various types of solid cancers.

Since CTC are found in very low frequency within the human bloodstream their reliable isolation and detection demands methodologies with high sensitivity and specificity. In recent years a variety of commercially available assays have been developed to detect CTC, including the US Food and Drug Administration (FDA)-cleared CellSearch^®^system (Veridex, Raritan, NJ, USA) [12-15], while new areas of research are directed towards developing novel assays for CTC molecular characterization.

Molecular assays for the detection and molecular characterization of CTC are very suitable for routine applications in the clinical laboratory setting, provided that they are robust and specific. Molecular assays are highly sensitive, easy to perform, detect viable CTC, have the advantage of *in silico *design, and can be easily automated and subjected to internal and external quality control systems. Another major advantage of molecular assays for CTC detection is the flexibility they offer, especially in a multiplex format, where we can significantly reduce the amount of precious CTC sample, as well as the time and cost of analysis. Several mRNA markers have been used for RT-PCR-based detection of CTC. Quantification of these mRNAs is essential to distinguish normal expression in blood from that due to the presence of CTC. Few molecular markers provide adequate sensitivity individually, but combinations of markers may offer better sensitivity for CTC detection.

The first identification of distinct molecular profiles of CTC has shown that detection of the expression of different genes can increase the CTC positivity of breast cancer patients and provide additional information concerning the clinical management of patients [16]. Since then a variety of multiplex RT-PCR assays for the molecular characterization of CTC have been developed, all targeting different mRNA transcripts [17-21]. Molecular profiling of low numbers of CTC, in positively immune-magnetically selected CTC [21-23] or even in a high background of leukocytes [18] is now feasible and shows promise for further studies on the clinical relevance of their molecular characterization. Molecular profiling of CTC at the single cell level has also been reported [24]. Recently our group developed a multiplex RT-qPCR assay for the quantification of *CK-19, HER-2, MAGE-A3 *and *PBGD *transcripts in early and metastatic breast cancer patients [21]. Another molecular assay, that is also commercially available, the AdnaTest BreastCancer™ (Alere, San Diego, CA, USA) is based on the isolation of CTC by immunomagnetic beads labeled with antibodies against MUC-1 and epithelial cell adhesion molecule (EpCAM), followed by multiplex RT-PCR for *GA 733-2, MUC1, HER-2 *and *beta actin *[11,25].

However, the existence of numerous technical approaches for enumeration and molecular characterization of CTC make the assessment of CTC as a routine procedure for the clinical management of breast cancer patients very complicated. Many questions still remain unanswered regarding the optimal method to enumerate and characterize CTC and the path to regulatory and general clinical acceptance of technology platforms currently under development [26]. Despite the numerous methods for CTC detection a major question that arises is whether these assays give the same information when analyzing the same clinical samples.

Standardization of assays used for the detection of CTC is essential before their use in clinical practice. Towards this direction, comparison studies between different analytical methodologies for CTC detection and molecular characterization are urgently needed [27-29]. So far, comparison studies have revealed distinct variations in the detection rates for the presence of CTC between different analytical platforms [13]. Many research groups have compared the FDA-cleared CellSearch platform with other CTC detection systems [27,30,31] and have shown discrepancies, possibly explained by the different approaches used for capturing and analyzing CTC.

The aim of our study was to compare three different molecular assays for the detection and molecular characterization of CTC in primary and metastatic breast cancer: a) a RT-qPCR assay that detects CTC through the expression of *CK-19 *[32]; b) a multiplex RT-qPCR assay that detects CTC through the expression of *CK-19, HER-2, MAGE-A3*, and *PBGD *[21]; and c) a commercially available molecular assay (AdnaTest BreastCancer™) that detects CTC through the expression of *GA733-2, MUC-1, HER-2 *and *beta actin *[22]. Our main question was whether these molecular assays give comparable results for the same samples in terms of CTC positivity or negativity, based on the fact that the molecular markers selected are different. In order to get a more precise comparison, we performed our study using the same cDNAs, to exclude all errors in the pre-analytic variables, such as sample isolation, sample volume, logistics and storage conditions, as well as important analytical variables, such as CTC isolation methodology, RNA isolation, and cDNA preparation steps [26].

## Materials and methods

### Patients

In total, 254 patients with operable breast cancer and 51 patients with verified metastasis were enrolled in the study. All patient samples were collected at the Department of Obstetrics and Gynecology in the University Hospital of Essen, Germany. Patients with primary breast cancer were enrolled from March 2007 until March 2009. Characteristics for primary breast cancer patients at the time of diagnosis are shown in Table 1. The majority of patients had small tumors and 51% were node-negative. Most patients had ductal breast cancer. Moderately and poorly differentiated tumors were predominant. A total of 179 of 254 (70%) patients were estrogen receptor (ER)-positive and 175 of 254 (69%) patients were progesterone receptor (PR)-positive. Human epidermal growth factor receptor 2 (HER2) expression was present in 38 of 254 patients (15%). Patients with metastatic breast cancer were enrolled from November 2006 until October 2009. The patient eligibility criteria were as follows: age ≥18 years; measurable or evaluable metastatic breast cancer; predicted life expectancy ≥2 months; Eastern Cooperative Oncology Group (ECOG) scores for performance status of 0 to 2; no severe uncontrolled co-morbidities or medical conditions; and no second malignancies. Patients had either a relapse of breast cancer diagnosed years before and were to start chemotherapy or a documented progressive breast cancer before receiving a new endocrine, chemo- or experimental therapy. Prior adjuvant treatment, radiation or any other treatment of metastatic disease were permitted. Exclusion criteria were other malignancies except breast cancer. Most patients had visceral and non-visceral metastasis. The chemotherapeutic adjuvant treatment mostly contained anthracyclines and taxanes. Patients with metastatic tumors of the breast received different chemotherapeutic treatments including anthracyclines, taxane, vinorelbine and 5-fluorouracil (5-FU). Most patients were extensively pre-treated before starting the collection of the blood samples. Nearly all patients with HER2 3+ tumors received trastuzumab in the metastatic setting. A group of 30 healthy female blood donors was used as control. All specimens were obtained after written informed consent and collected using protocols approved by the institutional review board (05/2856).

**Table 1:** CTC detection and clinical characteristics of early breast cancer patients

Patients enrolled		AdnaTest			*CK19*			Multiplex		
	All number (%)	Positive number (%)	Negative number (%)	*P*^a^	Positive number (%)	Negative number (%)	*P*^a^	Positive number (%)	Negative number (%)	*P*^a^
	254 (100)	42 (16.5)	212 (83.5)		36 (14.2)	218 (85.8)		58 (22.8)	196 (77.2)	
Age, years				0.398			0.383			0.614
≥ 61	130 (51.2)	24 (18.5)	106 (81.5)		16 (12.3)	114 (87.7)		28 (21.5)	102 (78.5)	
< 61	124 (48.8)	18 (14.5)	106 (85.5)		20 (16.1)	104 (83.9)		30 (24.2)	94 (75.8)	
Staging				0.827			0.343			
Tx	1 (0.39)	0 (0)	1 (100)		0 (0)	1 (100)		0 (0)	1 (100)	
is	8 (3.15)	2 (25.0)	6 (75.2)		0 (0)	8 (100)		1 (12.5)	7 (87.5)	
To	6 (2.36)	1 (16.7)	5 (83.3)		0 (0)	6 (100)		2 (33.3)	4 (66.7)	
I/II	206 (81.1)	29 (14.1)	177 (85.9)		34 (16.5)	172 (83.5)		48 (23.3)	158 (76.7)	
III/IV	19 (7.48)	4 (21.1)	15 (78.9)		1 (5.26)	18 (94.7)		4 (21.0)	15 (79.0)	
Unknown	14 (5.51)									
Grading				0.386			0.822			
1	45 (17.7)	5 (11.1)	40 (88.9)		6 (13.3)	39 (86.7)		12 (26.7)	33 (73.3)	0.816
2	105 (41.3)	19 (18.1)	86 (81.9)		17 (16.2)	88 (83.8)		23 (21.9)	82 (78.1)	
3	83 (32.7)	10 (12.1)	73 (88.0)		11 (13.2)	72 (86.8)		19 (22.9)	64 (77.1)	
Unknown	21 (8.37)									
Lymph nodes										
N0	130 (51.2)	17 (13.1)	113 (86.9)	0.099	23 (17.7)	107 (82.3)	0.450	31 (23.8)	99 (76.2)	0.871
N1	54 (21.3)	9 (16.7)	45 (83.3)		6 (11.1)	48 (88.9)		15 (27.8)	39 (72.2)	
N2	15 (5.91)	3 (20.0)	12 (80.0)		2 (13.3)	13 (86.7)		3 (20.0)	12 (80.0)	
N3	6 (2.36)	3 (50.0)	3 (50.0)		2 (33.3)	4 (66.7)		1 (16.7)	5 (83.3)	
Unknown	49 (19.3)									
Histology										
Ductal	175 (68.9)	23 (13.1)	152 (86.9)	0.251	23 (13.1)	152 (86.9)	0.201	39 (22.3)	136 (77.7)	
Lobular	35 (13.8)	7 (20.0)	28 (80.0)		9 (25.7)	26 (74.3)		12 (34.3)	23 (65.7)	
Ductal/Lobular	5 (1.97)	2 (40.0)	3 (60.0)		1 (20.0)	4 (80.0)		0 (0)	5 (100)	
Other	24 (9.45)	5 (20.8)	19 (79.2)		2 (8.3)	22 (91.7)		4 (16.7)	20 (83.3)	
Unknown	15 (5.91)									
ER				0.553			0.275			0.581
Positive	179 (70.5)	28 (15.6)	151 (84.4)		29 (16.2)	150 (83.8)		40 (22.3)	139 (77.7)	
Negative	58 (22.8)	11 (19.0)	47 (81.0)		6 (10.3)	52 (89.7)		15 (25.9)	43 (74.1)	
Unknown	17 (6.7)									
PR				0.474			0.184			0.361
Positive	175 (68.9)	27 (15.4)	148 (84.6)		29 (16.6)	146 (83.4)		38 (21.7)	137 (78.3)	
Negative	62 (24.4)	12 (19.4)	50 (80.6)		6 (9.7)	56 (90.3)		17 (27.4)	45 (72.6)	
Unknown	17 (6.7)									
HER2							0.725			0.397
Positive	38 (15.0)	7 (18.4)	31 (81.6)	0.639	5 (13.2)	33 (86.8)		11 (29.0)	27 (71.0)	
Negative	195 (76.8)	30 (15.4)	165 (84.6)		30 (15.4)	165 (84.6)		44 (22.6)	151 (77.4)	
Unknown	21 (8.27)									

^a^Chi-square test. ER, estrogen receptor; HER2, human epidermal growth factor receptor 2; PR, progesterone receptor.

### CTC isolation

Blood samples were taken from all patients and analyzed for CTC using the commercially available AdnaTest BreastCancer™ (Alere) kit which enables the immunomagnetic enrichment of tumor cells *via *epithelial and tumor associated antigens. Blood (2 × 5 ml with addition of ethylenediaminetetraacetic acid (EDTA)) was collected for isolation of CTC using the AdnaCollect blood collection tubes (Alere), stored at 4°C and further processed within 48 hours. Blood samples were incubated with a ready-to-use antibody mixture (against EpCAM and MUC1) according to the manufacturer's instructions and the labeled cells were extracted by a magnetic particle concentrator as previously described [11,22,25].

### RNA isolation from CTC and cDNA synthesis

Isolation of mRNA from lysed, enriched cells was performed with the Dynabeads mRNA DIRECT™ Micro Kit (Dynal, Karlsruhe, Germany) according to the manufacturer's instructions. Sensiscript^® ^Reverse Transcriptase (QIAGEN, Hilden, Germany) was used for reverse transcription because of its high sensitivity as previously described [11,22,25]. All cDNA samples were first checked for their quality using as control genes both *beta-actin *and *PBGD*. Only samples that were positive for both control genes were enrolled in this study. More specifically, we initially evaluated the quality of 541 cDNA samples, but only 305 cDNAs were positive and further used. All these samples were collected and analyzed immediately by the AdnaTest BreastCancer™ kit. However, many cDNAs, that we would have liked to use for *CK-19 *RT-qPCR and multiplex RT-qPCR were found to be degraded and of very pure quality. This is mainly due to the long time that these cDNA samples were kept at -20°C (for more than two years).

### CTC detection and molecular characterization

#### *CK-19 *RT-qPCR

A singleplex RT-qPCR was performed for *CK-19 *in the LightCycler 1.5 (Roche, Mannheim, Germany) as previously described [32]. The amplification reaction mixture contained 2 μL of the PCR Synthesis Buffer (5Χ), 1 μL of MgCl_2 _(25 mM), 0.2 μL dNTPs (10 mM), 0.15 μL BSA (10 μg/μL), 0.1 μL Hot Start DNA polymerase (HotStart, 5 U/μL, Promega, Madison, USA), 0.5 μL of each primer (10 μΜ), 1.0 μL of each hydrolysis probe (3 μM) and sterile H_2_O (added to 10 μL final volume). Cycling conditions were: 95°C for 3 minutes; 45 cycles of 95°C for10 seconds, annealing at 55°C for 20 seconds and extension at 72°C for 20 seconds.

#### Multiplex RT-qPCR

Multiplex RT-qPCR was performed for *CK-19, HER-2, MAGE-A3 *and *PBGD *was used as control gene as previously described [21]. Multiplex RT-qPCR reactions were performed in the LightCycler 2.0 (Roche). The amplification reaction mixture contained 2 μL of the PCR Synthesis Buffer (5Χ), 2.5 μL of MgCl_2 _(25 mM), 0.4 μL dNTPs (10 mM), 0.8 μL BSA (10 μg/μL), 0.4 μL Hot Start DNA polymerase (HotStart, 5 U/μL, Promega), 1 μL of all eight primers (10 μΜ for each), 0.5 μL of all eight dual hybridization probes (4 μM for each) and sterile H_2_O (added to 10 μL final volume). Cycling conditions were: 95°C for 3 minutes; 45 cycles at 95°C for 10 seconds, annealing at 60°C for 60 seconds and extension at 72°C for 30 seconds.

#### AdnaTest BreastCancerDetect

The AdnaTest BreastCancerDetect kit was used for the detection of *HER-2, MUC1 *and *GA733-2 *(*EpCAM*) in all cDNAS. PCR was performed with the HotStarTaq Master Mix (QIAGEN). *Beta-actin *was used as control gene. The thermal profile used was as follows: After a 15 minute denaturation at 95°C, 35 cycles of PCR were carried out by denaturation at 94°C for 60 seconds, annealing/extension at 60°C for 60 seconds and elongation for 60 seconds at 72°C. Subsequently, termination of the reaction was carried out at 72°C for 10 minutes followed by storage of the samples at 4°C. The primers generated fragments of the following sizes: *GA733-2*:395 bp, *MUC1*:293 bp, *HER-2*:270 bp, and *β-actin*:114 bp. Visualization of PCR fragments was carried out with a 2100 Bioanalyzer (Agilent Technologies, Santa Clara, CA, USA) using the DNA 1000 LabChips and the Expert Software Package (version B.02.03.SI307). The test was considered as CTC positive if a PCR fragment of at least one tumor-associated transcript (*MUC-1, GA773-2 *or *HER-2*) and a fragment of *β-actin *was clearly detected (peak concentration of > 15 ng/ul) in both blood samples. According to previous studies, this assay is highly sensitive as it can detect two cells per 5 ml of blood, and highly specific (95%) [11,22,25].

### Statistical analysis

The chi square test was used for the evaluation of concordance in the early breast cancer setting while in the verified metastasis group, where a lower number of cases was studied, we performed the Fischer's exact test. We have also used the Kappa test in all cases to evaluate the agreement between these molecular methods [33].

The outline of the whole experimental design is given in Additional file 1 as a figure.

## Results

### *CK-19 *RT-qPCR

This assay has already been analytically validated in detail in our previous studies [6,32] and has been shown to give results of clinical significance when applied in early breast cancer and CTC were isolated from 20 mL peripheral blood [7,8,32]. In the present study, in the group of early breast cancer patients, CTC were isolated according to the AdnaTest BreastCancer™ system from 2 × 5 mL peripheral blood and detected in 36/254 patients by *CK-19 *RT-qPCR (14.2%), while in the group of 51 patients with overt metastasis, CTC were detected in 21 patients (41.2%) (Table 2). In the group of 30 healthy individuals tested, one was found positive for *CK-19 *(3.3%).

**Table 2:** Comparison between *CK19 *RT-qPCR, multiplex RT-qPCR and AdnaTest for the detection of CTC in breast cancer

Molecular assay	*CK19 *RT-qPCR		Multiplex- RT-qPCR		Total
**Early breast cancer, (number = 254)**					

AdnaTest	**Positive**	**Negative**	**Positive**	**Negative**	**Total (%)**
Positive	4	38	5	37	42 (16.5)
Negative	32	180	53	159	212 (83.5)
Total (%)	36 (14.2)	218 (85.8)	58 (22.8)	196 (77.2)	254
Kappa test	poor agreement: Kappa = -0.059 (P = 0.344)		poor agreement: Kapp = -0.114 (P = 0.065)		
Concordance (%)	184 (72.4), *P*^a ^**= **0.344		164 (64.6), *P^a ^***= **0.065		

**Verified metastasis, (number = 51)**					

AdnaTest	**Positive**	**Negative**	**Positive**	**Negative**	**Total (%)**
Positive	17	11	16	12	28 (54.9)
Negative	4	19	4	19	23 (45.1)
Total (%)	21 (41.2)	30 (58.8)	20 (39.2)	31 (60.8)	51
Kappa test	Moderate agreement: Kappa = 0.422 (*P *= 0.002),		Fair agreement: Kappa = 0.386 (*P *= 0.004)		
Concordance (%)	36 (70.6), *P*^a ^**= **0.002		35 (68.6), *P*^a ^**= **0.004		

^a^chi-square test, kappa test; 95% CI (0.504, 0.848). The same result was found when we used the chi-square test (Table 2).

### Multiplex RT-qPCR

This assay has also been analytically validated in detail in our previous study where CTC were also isolated from 20 mL peripheral blood [21]. The multiplex RT-qPCR was considered positive if a PCR fragment of at least one tumor-associated transcript (*CK-19, MAGE-A3 *and *HER-2*) was detected. In the present study, in the group of early breast cancer patients, CTC were isolated according to the AdnaTest BreastCancer™ system from 2 × 5 mL peripheral blood and were detected in 58/254 (22.8%) by multiplex RT-qPCR (Table 2). As can be seen in Table 3, *CK-19 *was positive in 25/254 (9.8%), *MAGE- A3 *in 12/254 (4.7%) and *HER-2 *in 28/254 *(*11.0%) patients. In the group of patients with verified metastasis, CTC were detected in 20/51 (39.2%) (Table 2) with positive expression rates of 18/51 (35.3%) for *CK-19*, of 4/51 (7.8%) for *MAGE-A3 *and 8/51 (15.7%) for *HER-2 *(Table 3). In a total of 30 healthy individuals tested, 6.6% were found to be positive by the multiplex RT-qPCR for *CK-19, HER-2, MAGE-A3 *and *PBGD*.

**Table 3:** Comparison between *CK19 *RT-qPCR, multiplex RT-qPCR and AdnaTest for the molecular characterization of CTC in breast cancer.

		Multiplex RT-qPCR						Singleplex RT-qPCR	
AdnaTest/		***CK19***		***HER-2***		***MAGE A3***		***CK19***	
		
		**Pos**.	**Neg**.	**Pos**.	**Neg**.	**Pos**.	**Neg**.	**Pos**.	**Neg**.

		**Early breast cancer, (number = 254)**							

*GA733-2*	Positive	1	8	0	9	0	9	2	7
	Negative	24	221	28	217	12	233	34	211
	Concordance (%)	87.4	*P*^a ^= 0.897	85.4	*P*^a ^= 0.284	91.7	*P*^a ^= 0.496	83.9	*P*^a ^= 0.481
*MUC-1*	Positive	0	14	1	13	0	14	1	13
	Negative	24	215	27	213	12	228	35	205
	Concordance (%)	84.6	*P*^a ^= 0.213	84.3	*P*^a ^= 0.633	89.8	*P*^a ^= 0.391	81.1	*P*^a ^= 0.438
*HER-2*	Positive	2	24	2	24	0	26	2	24
	Negative	23	205	26	202	12	216	34	194
	Concordance (%)	81.5	*P*^a ^= 0.698	80.3	*P*^a ^= 0.567	85	*P*^a ^= 0.231	77.2	*P*^a ^= 0.317
		
		**Verified metastasis, (number = 51)**							
		
*GA733-2*	Positive	**14**	**6**	6	14	3	17	**15**	**5**
	Negative	**4**	**27**	2	29	1	30	**6**	**25**
	Concordance (%)	**80.4**	***P*^b ^< 0.001**	68.6	*P*^b ^= 0.045	64.7	*P*^b ^= 0.287	**78.4**	***P*^b ^= 0.000**
*MUC-1*	Positive	**14**	**9**	6	17	3	20	**14**	**9**
	Negative	**4**	**24**	2	26	1	27	**7**	**21**
	Concordance (%)	**74.5**	*P***^b ^= 0.001**	62.7	*P*^b ^= 0.119	58.8	*P*^b ^= 0.316	**68.6**	**P^b ^= 0.012**
*HER-2*	Positive	**11**	**7**	5	13	1	17	**13**	**5**
	Negative	**7**	**26**	3	30	3	30	**8**	**25**
	Concordance (%)	**72.6**	***P*^b ^= 0.006**	68.6	*P*^b ^= 0.112	60.8	*P*^b ^= 1	**74.5**	***P*^b ^= 0.001**

^a^chi-square test; ^b^Fisher's exact test; *P *< 0.05 is considered as significant.

### AdnaTest BreastCancer™

The AdnaTest BreastCancer™ assay is considered positive if a PCR fragment of at least one transcript is detected at a concentration of 0.15 ng/μl or higher. Using these criteria, all 30 healthy individuals tested were found to be negative, while in the group of early breast cancer patients, CTC were detected in 42/254 patients (16.5%) (Table 2) with the positive expression rates of 3.5% for *GA733-2*, 5.5% for *MUC-1 *and 10.2% for *HER-2 *(Table 3). In the group of patients with verified metastasis, CTC were detected in 28/51 patients (54.9%) (Table 2), with *GA733-2 *detected in 20/51 (39.2%), *MUC-1 *in 23/51 (45.1%) and *HER-2 *in 18/51(35.3%) patients (Table 3).

### Comparison between the three molecular assays for CTC detection

When the same target (*CK-19*) was detected in the same cDNAs with the same set of primers and probes there was a very good concordance between singleplex RT-qPCR and multiplex RT-qPCR. In early breast cancer, where the number of CTC and thus the number of mRNA transcripts are very low, we found a concordance for 223/254 (87.8%, *P *= 0.000) of all samples tested while in the verified metastasis setting the concordance was almost perfect, for 48/51 (94.1%, *P *= 0.000) of all samples tested (Table 4).

**Table 4:** Comparison between *CK-19 *RT-qPCR, and multiplex RT-qPCR for *CK-19 *detection

Molecular assay	*CK-19 *RT-qPCR			
***CK-19 multiplex *RT-qPCR**	**Early breast cancer, (number = 254)**		**Verified metastasis, (number = 51)**	
	
	**Positive**	**Negative**	**Positive**	**Negative**

Positive	15	10	18	0
Negative	21	208	3	30
Total (%)	36 (14.2)	218 (85.8)	21 (41.2)	30 (58.8)
Kappa test	Moderate agreement: Kappa = 0.425 (*P *= 0.000) Pa = 0.000P = = Pp(P = 0.000)		Almost perfect agreement: Kappa = 0.876 (*P *= 0.000)	
Concordance (%)	223 (87.8), **P^a ^= 0.000**		48 (94.1), **P^a ^= 0.000**	

^a^Chi-square test.

**Figure 1:**
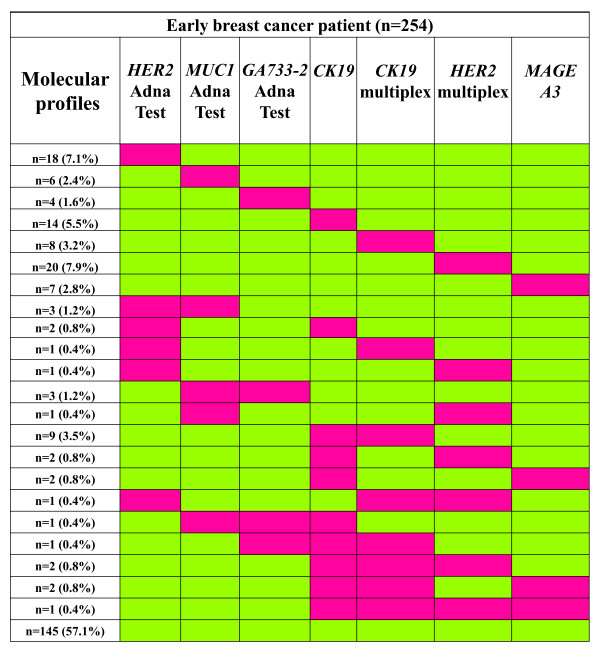
**Molecular profiling of CTC in early breast cancer (*n *= 254)**. Red and green indicates positive and negative detection, respectively.

In early breast cancer, we found an agreement between the AdnaTest BreastCancer™ and *CK-19 *RT-qPCR in 184/254 (72.4%) of the cases. However, the correlation concerning the CTC positivity between these two assays was not significant (*P *= 0.344), since there were 32 samples that were found positive for CTC by the *CK-19 *assay but negative by the AdnaTest and 38 samples found positive for CTC by the AdnaTest but negative by the *CK-19 *assay. The concordance between the AdnaTest and multiplex RT-qPCR in this group of patients was 64.6%. There was no significant correlation (*P *= 0.065) in this case as well, since there were 53 samples found positive for CTC by the multiplex RT-qPCR assay but negative by the AdnaTest and 37 samples found positive for CTC by the AdnaTest but negative by the multiplex RT-qPCR assay (Table 2). In this group, all combinations found for each molecular target are shown in Figure 1.

**Figure 2:**
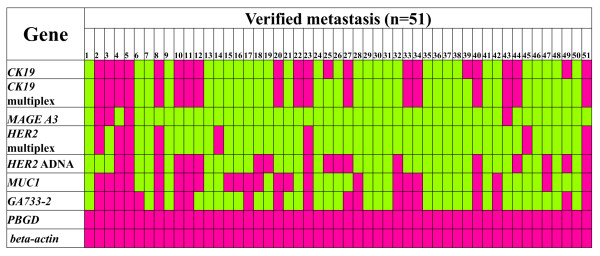
**Molecular profiling of CTC in verified metastasis (*n *= 51)**. Red and green indicates positive and negative detection, respectively.

In the group of patients with verified metastasis, we found an agreement between the AdnaTest and *CK-19 *RT-qPCR in 36/51 (70.6%) of cases. The correlation concerning the CTC positivity between these two assays was significant (*P *= 0.002), since there were only four samples that were found positive for CTC by the *CK-19 *assay but negative by the AdnaTest and 11 samples found positive for CTC by the AdnaTest but negative by the *CK-19 *RT-qPCR. In all other cases these two assays gave similar results. Between the AdnaTest and multiplex RT-qPCR, the concordance rate in the group of patients with verified metastasis was 68.6%. There was a significant correlation (*P *= 0.004) in this case as well, since there were only four samples that were found positive for CTC by the multiplex RT-qPCR but negative by the AdnaTest and 12 samples found positive by the AdnaTest but negative by the multiplex RT-qPCR (Table 2). In all the other cases, these two assays gave similar results as well. In this group, the comparison results for each individual sample are shown in a heatmap (Figure 2).

### Comparison of AdnaTest BreastCancer™ and multiplex RT-qPCR for *HER-2 *expression in CTC

*HER-2 *is the only common target between the AdnaTest BreastCancer™ and multiplex RT-qPCR; however, different primers targeting completely different sequence areas are used by these two molecular assays for evaluating *HER-2 *expression.

Concerning *HER-2 *expression in CTC there was no significant correlation (*P *= 0.567) between multiplex RT-qPCR and the AdnaTest, since there were 26 samples that were found positive for *HER-2 *in CTC by the multiplex RT-qPCR assay but negative by the AdnaTest and 24 samples found positive for *HER-2 *in CTC by the AdnaTest but negative by the multiplex RT-qPCR (Table 3). As can be seen in Table 3, in the early breast cancer group, there were only two samples that were found positive for *HER-2 *in CTC both by the multiplex RT-qPCR and by the AdnaTest, while 202 samples were found negative by both assays.

In the group of patients with verified metastasis (Table 3), there were only five samples found to be positive for *HER-2 *in CTC, both by the multiplex RT-qPCR and by the AdnaTest, while 30 samples were found to be negative by both assays. There was no significant correlation (*P *= 0.112), since three samples were found positive for *HER-2 *in CTC by the multiplex RT-qPCR but negative by the AdnaTest and 13 samples were found positive for *HER-2 *in CTC by the AdnaTest but negative by the multiplex RT-qPCR. *HER-2 *comparison results for each individual sample can be seen in Figure 2.

We also had the information on HER-2 expression in the primary tumors for 233 of these samples. The AdnaTest detected *HER-2 *expression in CTC in 24 patients, but HER-2 status of the primary tumor was positive and concordant in only four patients, negative and discordant in 20, while in 175 cases *HER-2 *expression was not detected in either the primary tumor or in CTC. The multiplex RT-qPCR detected *HER-2 *expression in CTC in 27 patients, but HER-2 status of the primary tumor was positive in only six of these patients, negative and discordant in 21, while in 174 cases HER-2 expression was not detected in either primary tumor or in CTC (Additional file 2).

## Discussion

Quality control is an important issue for the clinical use of CTC analysis, and standardization of CTC detection and characterization methodologies are important for the incorporation of CTC into prospective clinical trials testing their clinical utility [26]. Moreover, the analytical assays used and the degree of their validation will be critical to establishing a common set of criteria describing CTC [26]. Despite the fact that most CTC assays are highly specific and sensitive, there are not so many extensive studies especially designed to compare their efficacy when using the same clinical samples. This is an important issue for their clinical use since, especially in early disease, differences in analytical sensitivity between CTC assays play a very critical role. Among the recommendations described in a recent report by the AACR-FDA-NCI Cancer Biomarkers Collaborative, inter-laboratory and intra-laboratory comparison studies for the same samples are urgently needed [34].

So far, the main technology where quality control issues have been addressed in the field of CTC assays is the CellSearch [27,30,35,36]. In a recent comparison study between the CellSearch and AdnaTest BreastCancer™ assays, concordant results regarding HER-2 positivity were obtained only in 50% of the patients [37]. Another recent study directly compared three techniques for detecting CTC in blood samples: the CellSearch, the AdnaTest BreastCancer™ and an in-house RT-qPCR assay and found a substantial variation in the detection rates of CTC in blood from breast cancer patients [38]. The results of the DETECT trial which was designed to compare directly the prognostic value of two commercially available CTC assays in metastatic breast cancer have shown that the prognostic relevance of CTC detection depends on the test method, and that the CellSearch assay is superior to the AdnaTest BreastCancer™ assay in predicting clinical outcome [39]. However, another study has revealed that the AdnaTest BreastCancer™ has equivalent sensitivity to that of the CellSearch in detecting two or more CTC [40]. Moreover, recent studies, especially in early breast cancer, have also shown a superior sensitivity of molecular assays with respect to the CellSearch [11,21,25,28]. In most of these comparison studies, discrepancies found between the different assays were partly explained by the fact that these molecular assays were using different capture technologies for CTC isolation and very different detection systems.

In the present study, we compared three molecular assays for the detection and molecular characterization of CTC after excluding all errors in the pre-analytic variables, such as sample isolation, sample volume, logistics and storage conditions, as well as important analytic variables, such as CTC isolation methodology, RNA isolation, and cDNA preparation steps [26]. In this way, we evaluated the effect of using different molecular transcripts on CTC detection.

When the same target (*CK-19*) was detected in the same cDNAs with the same set of primers and probes there was a very good concordance between singleplex RT-qPCR and multiplex RT-qPCR. Slight discrepancies in this case could possibly reflect differences in the probability of finding mRNA transcripts in these samples due to the very low expected CTC numbers (Poisson distribution).

When the same target (*HER-2*) was detected in the same cDNAs with a different set of primers, targeting different regions in the gene sequence, and by different detection systems, results were not statistically correlated. However, there was a concordance between findings for 80.3% of cases in early breast cancer and 68.6% of cases in patients with verified metastasis. In both cases the majority of the samples were found to be negative by both primers sets (79.5% in early and 58.8% in metastasis). There were discrepancies in 19.7% of early and 31.4% of metastasis cases, while a very small number of samples were found to be positive by both primer sets (Table 3). When the presence of CTC positivity was assessed based on completely different transcripts, as in the case of singleplex RT-qPCR for *CK-19 *versus AdnaTest BreastCancer™, or multiplex RT-qPCR versus AdnaTest BreastCancer™, there were discrepancies when the number of CTC was low, as in early breast cancer. On the contrary, in cases where the number of CTC was higher, as in verified metastasis, these assays gave comparable results even while targeting different transcripts. Especially *CK-19 *expression was significantly correlated in all cases when compared to transcripts detected by the AdnaTest BreastCancer™ (Table 3).

We now know that CTC are highly heterogeneous even in the same patient [24]. Based on this, we could possibly explain the discrepancies found between these molecular assays by the fact that they are targeting different gene transcripts in CTC. In this way these assays can give the same result for the presence or absence of CTC in a clinical sample, only in the case that all these targets are simultaneously present or absent in a sample. Another explanation for the lack of statistical correlation in early breast cancer could possibly be the very low tumor load in this case. In verified metastasis, where there is a higher tumor load, a statistical agreement was verified between these assays, and *CK-19 *expression correlated with *HER-2, GA733-2 *and *MUC-1*. According to our data, in early breast cancer the same cDNA samples could be considered positive for the presence of CTC by one molecular assay and negative by the other. This is, however, very critical, especially when important clinical decisions are going to be based on this information, such as the administration of trastuzumab [10]. It is also obvious that much higher discrepancies are expected when different blood volumes and different isolation systems for CTC are used.

As an example, the immunoselection procedure followed in this work (since we are handling samples isolated by the AdnaTest) has modified the CTC positivity as based on our previously reported *CK-19 *assay that is not using any immunoselection [6-8,10,32]. There is no doubt that a different population of cells is isolated by using these different procedures. It is completely different to analyze only EpCAM- and MUC1-positive cells with respect to a highly sensitive *CK-19 *detection among all peripheral blood mononuclear cells (PBMCs) without any influence of the EpCAM expression on these cells. Moreover, in our already published protocol without immunoselection we are using 20 mL of peripheral blood for CTC isolation in the adjuvant setting [6,32], whereas in this case we are using cDNAs from EpCAM-positive CTC isolated from 2 × 5 mL peripheral blood. This could also explain the lower rates in *CK-19 *positivity seen in this sample cohort, in respect to the positive rates that we have previously reported. This could be evaluated in a future study, where the same peripheral blood samples from a significant number of patients could be analyzed by these molecular assays starting from the CTC isolation step and not from the cDNA step, adding an extra level of variability.

Recent genetic analyses of paired samples from primary tumors and disseminated tumor cells have uncovered a bewildering genetic disparity, questioning the use of primary tumors as surrogates for the genetics of systemic cancer [41]. In the era of molecular therapies that build upon genetic defects of tumor cells, these data call for a direct diagnostic pathology of systemic cancer [41]. Towards this direction, molecular assays are high-throughput, robust, sensitive and highly specific for the molecular characterization of CTC. However, our data indicate the importance of CTC heterogeneity for their detection by different molecular assays. A universal internal and external quality control system both for CTC detection, enumeration and molecular characterization is urgently needed before their application in the clinic. Moreover, the most important aspect for the multitude of assays developed for the detection of CTC is the clinical relevance of the results obtained.

## Conclusions

Quality control is an important issue for the clinical use of CTC analysis, and standardization of CTC detection and characterization methodologies are important for the incorporation of CTC into prospective clinical trials testing their clinical utility Towards this direction, comparison studies between different analytical methodologies for CTC detection and molecular characterization are urgently needed, since standardization of assays is essential before their use in clinical practice. In the present study we compared three molecular assays for the detection and molecular characterization of CTC after excluding all errors in the pre-analytic variables, such as sample isolation, sample volume, logistics and storage conditions, as well as important analytic variables such as the CTC isolation methodology, RNA isolation, and cDNA preparation steps. In this way, we evaluated the effect of using different molecular transcripts on CTC detection. When the same target was detected in the same cDNAs with the same set of primers and probes there was a very good concordance between singleplex RT-qPCR and multiplex RT-qPCR. When the same target was detected in the same cDNAs with a different set of primers, targeting different regions in the same gene sequence (*HER-2*), and by different detection systems, results were not statistically correlated. When the presence of CTC positivity was assessed based on completely different transcripts, there were discrepancies when the number of CTC was low, as in early breast cancer. On the contrary, in cases where the number of CTC was higher, as in verified metastasis, these assays gave comparable results even while targeting different transcripts. Our data indicate the importance of CTC heterogeneity for their detection by different molecular assays.

## Abbreviations

bp: base pair; BSA: bovine serum albumin; *CK-19*: cytokeratin 19; CTC: circulating tumor cells; EpCAM: epithelial cell adhesion molecule; ER: estrogen receptor; *HER-2*: human epidermal growth factor receptor-2; PR: progesterone receptor; RT-qPCR: reverse transcription quantitative polymerase chain reaction.

## Competing interests

SKB is a consultant for AdnaGen AG, Langenhagen, Germany. The other authors declare that they have no competing interests.

## Authors' contributions

AS carried out the molecular analysis and drafted the manuscript. SKB carried out the AdnaTest molecular assays and helped to draft the manuscript. AM and CP carried out part of the molecular analysis. ESL conceived the study and participated in its design and coordination, and drafted the manuscript. All authors read and approved the final manuscript.

## Supplementary Material

Additional file 1 **Outline of the study**. Figure outlining the whole experimental design of the study of the study. Click here for file

Additional file 2 **Comparison of *HER-2 *between the primary tumor and CTC**. *HER-2 *expression in the primary tumor and CTCs for 233 of these samples, as evaluated by multiplex RT-qPCR and the AdnaTest (*n *= 233). Click here for file
