# Supplementary figures and images for: Comparison of three molecular assays for the detection and molecular characterization of circulating tumor cells in breast cancer
Source: Breast Cancer Res. 2013 Mar 7;15(2):R20. doi: 10.1186/bcr3395 (PMC3672668; doi:10.1186/bcr3395)

## Additional file 1

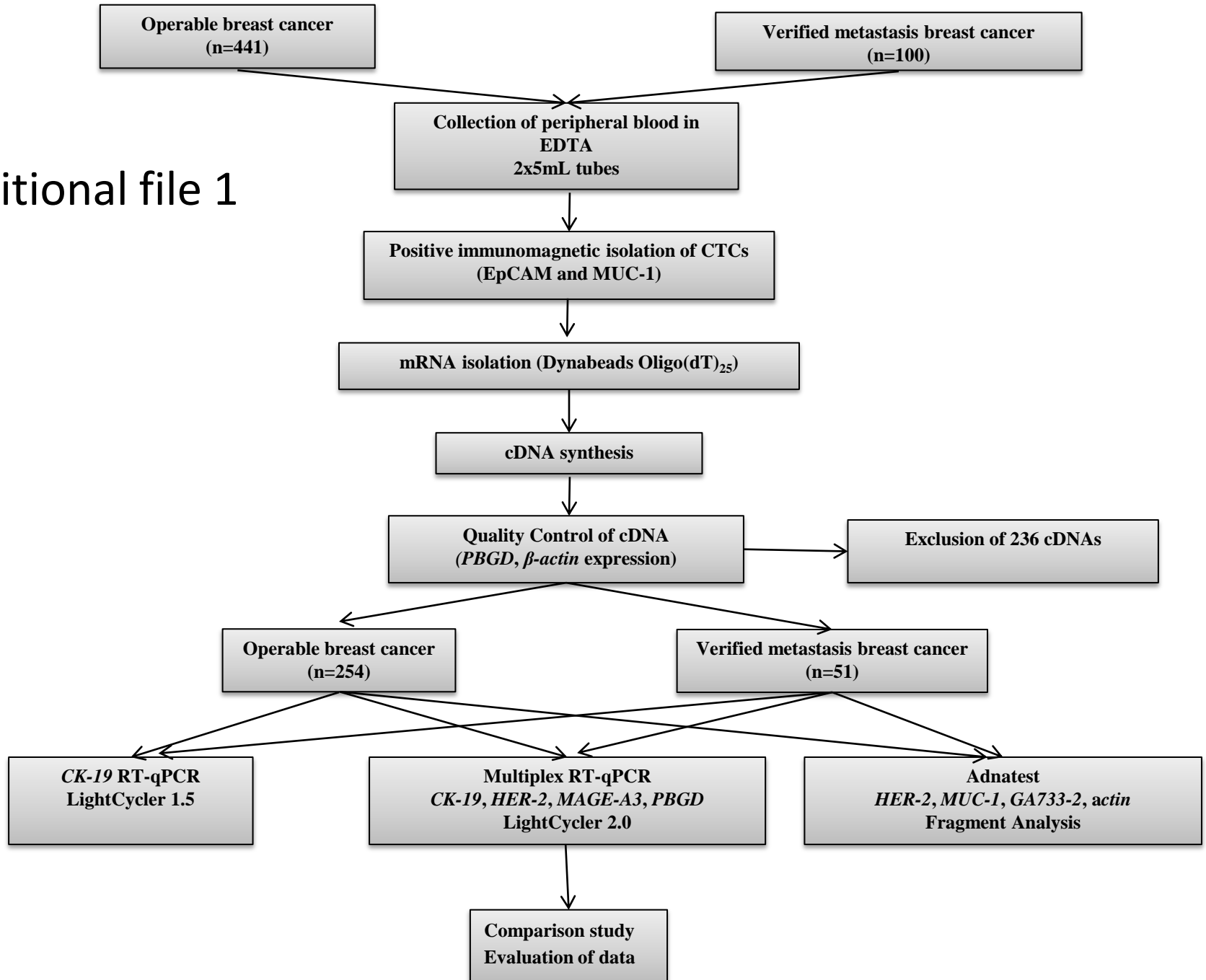

Supplement: Additional file 1 — Outline of the study. Figure outlining the whole experimental design of the study of the study. [file bcr3395-S1.PDF]
