# Supplementary material for: Comparison of three molecular assays for the detection and molecular characterization of circulating tumor cells in breast cancer
Source: Breast Cancer Res. 2013 Mar 7;15(2):R20. doi: 10.1186/bcr3395 (PMC3672668; doi:10.1186/bcr3395)
Supplement: Additional file 2 — Comparison of HER-2 between the primary tumor and CTC. HER-2 expression in the primary tumor and CTCs for 233 of these samples, as evaluated by multiplex RT-qPCR and the AdnaTest (n = 233). [file bcr3395-S2.DOCX]

**Additional File 2.** Comparison of *HER-2* between the primary tumor and CTC, as evaluated by multiplex RT-qPCR and the *AdnaTest* (n=233).

| Histology/Gene expression | *AdnaTest* | | Multiplex RT-qPCR | |
| --- | --- | --- | --- | --- |
| HER-2 | Positive | Negative | Positive | Negative |
| **Positive** | 4 | 34 | 6 | 32 |
| **Negative** | 20 | 175 | 21 | 174 |
| **Concordance** | 76.8% | P^a^=0.960 | 77.2% | P^a^=0.376 |
